# Supplementary material for: Evaluation of Zonulin Expression and Its Potential Clinical Significance in Glioblastoma
Source: Cancers (Basel). 2024 Jan 14;16(2):356. doi: 10.3390/cancers16020356 (PMC10814510; doi:10.3390/cancers16020356)
Supplement: Supplementary file 1 [file cancers-16-00356-s001.zip › cancers-2788726-supplementary.pdf]

Supplementary Table 1. Clinical, radiological and molecular features of patients included in the analysis of the circulating levels of zonulin.

Age		64.07 (SD=10.70)
Gender (male:female)		14:13
Karnofsky<70		3 (11.1%)
Contrast enhacement	Periferic	15 (55.6%)
	Heterogeneus	12 (44.4%)
Contrast enhancement volume (cc)		22.94 (SD=16.29)
Edema volume (cc)		53.19 (SD=32.83)
Necrosis volume (cc)		10.69 (SD=11.54)
Resection	Partial	8 (29.6%)
	Subtotal	5 (18.5%)
	Total	14 (51.9%)
Ki67 (%)		24.92 (SD=13.66)
MGMT methylation		15 (55.5%)
Zonulin (serum, ng/mL)		454.19 (SD=413.76)
Haptoglobin (serum, mg/dL)		208.83 (SD=85.42)

Supplementary table 2. Clinical, radiological and molecular features of patients included in the analysis of the zonulin expression in tumoral probes.

Age		65.33 (SD=11.87)
Gender (male:female)		10:11
Karnofsky<70		2 (9.5%)
Contrast enhacement	Periferic	8 (38.1%)
	Heterogeneus	13 (61.9%)
Contrast enhancement volume (cc)		19.76 (SD=14.19)
Edema volume (cc)		59.24 (SD=36.6)
Necrosis volume (cc)		8.46 (SD=9.48)
Resection	Partial	2 (9.5%)
	Subtotal	4 (19.0%)
	Total	15 (71.4%)
Ki67 (%)		27.4 (SD=19.53)
MGMT methylation		10 (47.6%)
Zonulin (Protein, from Western-Blot)		1245.23 (SD=1670.56)

Supplementary table 3. Comparison between groups of high vs. low haptoglobin (HP) circulating levels.

Variable		Low HP (n=13)	High HP (n=14)	p-value
Age (years)		63.8 (SD=10.06)	64.4 (SD=11.64)	0.923[a]
Gender (male:female)		6:7	8:6	0.706[b]
Karnofsky<70		-	3 (21.4%)	0.222[b]
Contrast enhacement	Periferic	7 (53.8%)	8 (57.1%)	0.863[b]
	Heterogeneus	6 (46.2%)	6 (42.9%)	
Contrast enhancement volume (cc)		25.79 (SD=19.15)	20.29 (SD=13.28)	0.593[a]
Edema volume (cc)		51.52 (SD=36.74)	54.74 (SD=30.08)	0.808[a]
Necrosis volume (cc)		9.13 (SD=12.28)	12.13 (SD=11.06)	0.286[a]
Resection	Partial	4 (30.8%)	4 (28.6%)	0.922[c]
	Subtotal	2 (15.4%)	3 (21.4%)	
	Total	7 (53.8%)	7 (50.0%)	
Ki67 (%)		21.82 (SD=12.46)	27.36 (SD=14.51)	0.410[a]
MGMT methylation		4 (33.3%)	11 (78.6%)	0.045[b]
Progression free survival (months)		4.5 [2.9 – 6.1]	15.0 [5.1 – 24.8]	0.108[d]

[a] Mann-Whitney U. [b] Fisher's Exact test. [c] Chi-Square. [d] Log-Rank test.

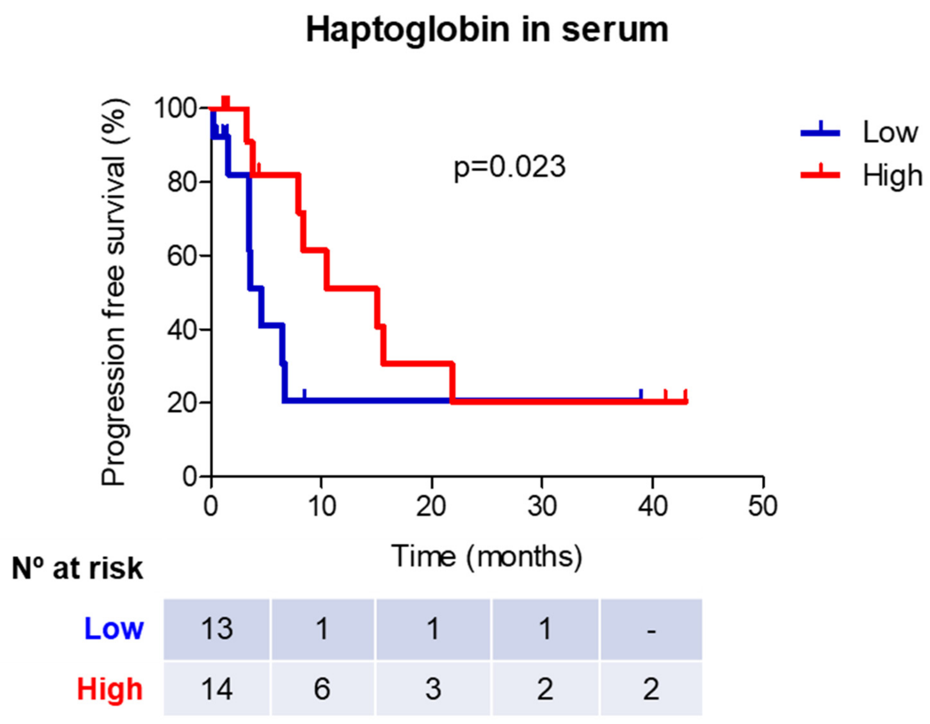

Supplementary figure 1. Survival analysis in different groups of haptoglobin (circulating levels).

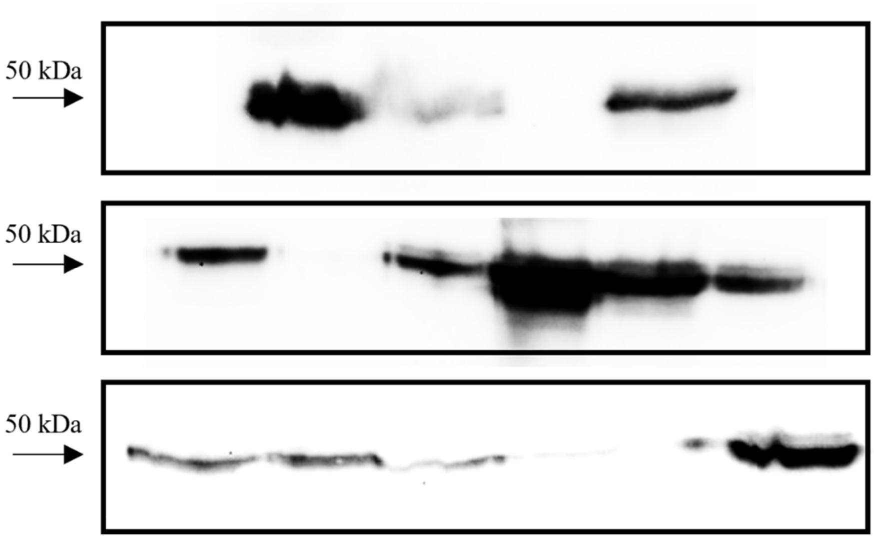

Supplementary figure 2. Examples of WB acquisitions for GB tumoral probes. Zonulin band corresponds to a MW of approximately 50 kDa.
